# Recent Major Transcriptomics and Epitranscriptomics Contributions toward Personalized and Precision Medicine

**DOI:** 10.3390/jpm12020199

**Published:** 2022-02-01

**Authors:** Ghada Mubarak, Farah R. Zahir

**Affiliations:** 1 Research Institute, Sidra Medicine, Doha, Qatar; gmubarak@sidra.org; 2 Department of Medical Genetics, University of British Columbia, Vancouver, BC V6H 3N1, Canada

**Keywords:** precision medicine, personalized medicine, cancer, cardiovascular disease, neurodevelopmental disorders, intellectual disability, autism spectrum disorder, transcriptomics, epitranscriptomics

## Abstract

With the advent of genome-wide screening methods—beginning with microarray technologies and moving onto next generation sequencing methods—the era of precision and personalized medicine was born. Genomics led the way, and its contributions are well recognized. However, “other-omics” fields have rapidly emerged and are becoming as important toward defining disease causes and exploring therapeutic benefits. In this review, we focus on the impacts of transcriptomics, and its extension—epitranscriptomics—on personalized and precision medicine efforts. There has been an explosion of transcriptomic studies particularly in the last decade, along with a growing number of recent epitranscriptomic studies in several disease areas. Here, we summarize and overview major efforts for cancer, cardiovascular disease, and neurodevelopmental disorders (including autism spectrum disorder and intellectual disability) for transcriptomics/epitranscriptomics in precision and personalized medicine. We show that leading advances are being made in both diagnostics, and in investigative and landscaping disease pathophysiological studies. As transcriptomics/epitranscriptomics screens become more widespread, it is certain that they will yield vital and transformative precision and personalized medicine contributions in ways that will significantly further genomics gains.

## 1. Introduction

Since microarray technology heralded the advent of the third era in medical diagnostics in the early “noughties”, clinical medical genetics has focused on genome-wide screens rather than targeted approaches. Shortly after chromosomal microarray analysis (CMA) was accepted and widely adopted as a first-tier diagnostic test in medical genetics [1], next generation sequencing (NGS) technologies exploded onto the scene. Indeed, the past decade has seen an acceleration in the development and implementation of a plethora of NGS based genomics screens that are ever-decreasing in cost, ever-increasing in diagnostic and clinical utility, and therefore, unsurprisingly, undergoing rapid expansion in utilization by diagnostic laboratories. The most common NGS screens involve interrogating the DNA sequence of the protein coding portion of the genome, termed whole exome sequencing (WES), followed by interrogating the entire whole genome, termed whole genome sequencing (WGS). These techniques and their reach in medical diagnostics have been extensively reported on and reviewed [2,3,4,5,6,7,8]. 

The latter half of the past decade, however, has given us a wide range of NGS-based screening methods other than WES and WGS. The variety and frequency of publication on the plethora of such “omics” approaches admittedly have even caused amusement as scientists and health care providers grapple to keep abreast of them. Indeed, tropes such as “other-omics” or “everything-omics” have commanded some popularity in various media [9]. Nevertheless, some of these “other-omics” have come into their own as robust sciences [10,11] that are now moving inevitably and one hopes, with very positive contribution, into the sphere of the clinic. 

In this paper, we overview the most relevant contributions of a major omics discipline—transcriptomics, and briefly touch on its continuum—epitranscriptomics—toward efforts in disease characterization and diagnostics as part of personalized and precision medicine initiatives (see Box 1: Comparing and contrasting personalized and precision Medicine). We focus our review on major advances in trascriptomics for three diseases: cancer, cardiovascular disease (CVD), and neurodevelopmental disorders (ND). They constitute the three disease areas where, in our estimation, the most significant developments in precision and/or personalized medicine have occurred. The recently emerging field of epitranscriptomics is necessarily dependent on the field of transcriptomics. Epitranscriptomics (as we detail in later sections) is the study of epi-modifications, i.e., chemical changes to the expressed gene transcript. Thus, advances in this field are intrinsically tied to advances in transcriptomics research. When considered via the lens of precision or personalized medicine, therefore of the three diseases we discuss for transcriptomics advances, we find the most notable contributions are in the ND domain. Hence, we only discuss epitranscriptomics for ND in depth.

Box 1 **Precision vs. personalized medicine.**

It is worth elucidating the distinction between precision medicine and personalized medicine, two terms that are often erroneously and confusingly used interchangeably:

Precision medicine’s main concern is to pinpoint the cause of disease or the particular aberration, be it a genomic variant, an environmental insult, or injury. Once the root cause is isolated, then treatment is focused to that nexus. The philosophy being that correcting the precise wrong will bring about clinical healing in the most efficient manner.

In contrast, personalized medicine’s main concern is to identify the cause of disease in a person and not consider the disease itself as a separate entity from the person. That is, it may be that a particular nexus of causation is identified—let us say, for example, a specific genetic aberration, but the point of emphasis is that this particular genetic aberration in this particular individual is causative. In other words, the context of the individual (genomic background, health metrics, environmental signatures, etc.) is taken into consideration and plays a significant role.

In practice, the results of a precision medicine and personalized medicine analysis may lead to the same conclusions. In fact, the difference between them can be confused, as they could include each other’s spheres of reference; often, a precision medicine effort to precisely pinpoint the genetic cause of a disease involves a discussion of the genomic background it presents on (i.e., personalized), and likewise a personalized medicine effort to look for a disease cause in a given individual will pinpoint a precise genetic variant (i.e., precision). However, the frame of reference and the philosophical basis of contemplation bears important distinction between the two.

## 2. Transcriptomics—A Key “Other-Ome”: An Introduction and Overview

### 2.1. Defining the “Ome”

Firstly, we must emphasize that when using the term “omic”, what is intended is that the whole is looked at rather than a part or a target. While there are reports that the first coining of the term “genomics” occurred in 1986 by Dr. Thomas Roderick during an informal meeting [12], the word “genome” was first reported in 1920 in a German publication by Hans Winkler, who stated “*I propose the expression Genom for the haploid chromosome set, which, together with the pertinent protoplasm, specifies the material foundations of the species…*” [13]. From this, was birthed the phenomena of terming every possible biological science where the whole is looked at as “something or the other -ome”! In fact, so ubiquitous was the trend at one time, that an interesting report speculates on the connection of this sound to a mantra invoking the divine [14]. It should also be noted, that while it may sound as if the suffix ”ome” has a Greek origin, no such reports exist. Nevertheless, the suffix has claimed as foundational a place as other medical terms with origin in the ancient Greek medical lexicon. Indeed, rightly so, as the immense impact of “omics” (omics being the assay/techniques/science that studies the relevant *ome*) science and clinical contributions to society in numerous ways testify. 

### 2.2. Transcriptomes and Transcriptomics

While the study of the whole complement of DNA sequence has been well established in personalized medicine, especially WES and WGS [11,15,16,17,18,19], transcriptomics is slowly but surely catching up. Transcriptomics refers to the study of the full complement of gene expression. This can be understood as the sum of the mRNAs in a cell or sample [20]. However, the major point to note is that the transcriptome includes all the product of transcription, sans inherent consideration of subsequent translation. In other words, while one can select only the mRNA for analysis, the transcriptome theoretically includes all types of RNAs produced. The vast majority of these are not mRNA. They include a diverse array of RNAs, collectively termed non-coding RNA (ncRNA), comprising several major RNA classes such as ribosomal RNA (rRNA), transfer RNA (tRNA), long non-coding RNA (lncRNA), micro-RNA (miRNA), etc. The above ncRNAs may be categorized in several ways, a useful grouping involves rRNAs and tRNAs together being termed “housekeeping RNAs”, while the other ncRNA classes are included in “regulatory RNAs” when it has been proven they are involved in cellular regulatory processes. 

The vast majority of the ncRNA pool is rRNA, which a cell produces in copious amounts as it forms the building material for ribosomes. rRNA from an isolate is generally depleted prior to sequencing as their abundance seriously impedes detecting signal from other RNAs [20], especially if mRNA is the objective of the assay. However, there are many classes of ncRNA other than rRNA, and they have recently been found to play an essential role in regulation of transcription (regulatory RNAs), as we elaborate on below. For a full review of all rRNA classes see [21,22]. 

Of the entire human genome, only ~2% is considered to be protein-coding gene sequence. However, ~70–80% of the genome is transcribed [23]. If one supposes that about half of protein-coding genes were to be transcribed in a given cell, and that 80% of the genome was represented in that cell’s transcriptome at a given time, fully 98.75% of the total transcripts are in fact ncRNA. Thus, while a transcriptomic assay is able to capture the entire complement of transcribed sequence, it remains up to the study design to determine which RNA component will be analyzed. Several transcriptomic studies are limited to mRNA alone, often loosely termed “gene expression” analysis [24]. However, others include specific RNA classes, such as lncRNA [25], sncRNA [26], miRNA [27], as examples. Therefore, while it is established that the transcriptome is a capture of RNA sequence, it is important that definitions are examined carefully when speaking of transcriptomes or transcriptomic profiling. In the next section, we will describe common transcriptome assays and how they have been used. 

### 2.3. Gene Expression Arrays 

The earliest transcriptomic assay was a complement to the earliest genomic assay—the microarray. Soon after the first successful CMA genomics screens were conducted, it did not take long to demonstrate CMA for the coding transcriptome, by simply manipulating an mRNA sample to convert it back to cDNA and using the same techniques as for genomic CMA. The earliest reported gene expression array studies [28,29] utilized this approach. As microarray technology became more mainstream, all the major microarray providers (Affymetrix^®^—ThermoFisher Scientific, California, USA, Agilent^®^—Agilent Technologies, California, USA, NimbleGen^®^—Roche NimbleGen, California, USA etc.) developed and marketed specialized microarray platforms for gene expression studies [28,30,31,32]. Illumina^®^, so well known for their NGS (next generation sequencing) sequencers, also entered the expression microarray field with its still popular Illumina BeadArray™ microarray (Illumina Inc., California, USA) [33]. Notably, all the above platforms are configured by default to mRNA expression analysis, as the technology is limited by only being able to interrogate products which will hybridize a pre-existing probe sequence manufactured and bonded to the microarray. Thus, in order to detect an expression signal, the sequence being looked for must already be known, and therefore, this meant that only mRNA (or more correctly, its complimentary cDNA sequence) was typically included. While more detailed reviews on microarray technology exist [34,35,36,37], for the purposes of this paper it is sufficient to note that a gene expression microarray experiment involved: isolation of mRNA from a given sample, conversion of the mRNA to cDNA, hybridization to microarray platform, and data analyses yielding a comparison of gene expression level between samples in case of two-color hybridization arrays, and comparison of gene expression level to a normal reference in case of one-color hybridization. This results in a differential gene expression (DEG) profile.

### 2.4. RNAseq

In contrast RNAseq, which is an NGS assay, is able to obtain a profile of transcript sans a pre-engineered substrate. Therefore, theoretically it is possible to obtain all transcript sequences, and this key advantage has led to RNAseq rapidly taking over microarray technology during the past decade, as the gene expression transcriptomic screen of choice. However, in practice, the over-abundance of rRNA in any total RNA isolate is a significant challenge. It is estimated that up to 85% of the total RNA isolate will be rRNA [38] and typically an rRNA depletion process and/or a pull down for mRNA by hybridizing its poly-A tail is first carried out prior to sequencing [38]. Thereafter, a variety of sample preparatory options are available for RNAseq. A thorough review of all the myriad RNAseq methods is beyond the scope of this paper, the interested reader is referred to useful reviews [39,40,41,42,43]. The type of sample preparation and type of sequencing technology plus complimenting bioinformatic processing, will determine what type of RNA profiling is obtained. Typically, DEG profiles are the most often generated, using short read sequencing technology. Nevertheless, as more sophisticated sample preparatory methods are developed and implemented, a growing number of studies are now providing information on lncRNA profiles, miRNA profiles, and a variety of other regulatory RNA profiles (for pertinent examples see Trivli, et al. [44], Dard-Dascot, et al. [45], and Beermann, et al. [46]). These efforts, along with concomitant large-scale epigenomic landscaping projects such as the ENCODE [47] and Roadmap project [for project suite of publications see http://www.roadmapepigenomics.org/publications/ (accessed on 15 December 2021)] are providing a fuller picture as to the structure and function of the transcriptome. Thus, the advent of NGS as a powerful and unbiased method to landscape and investigate the entire transcriptome has given immense yields for precision and personalized medicine as we overview next.

## 3. Transcriptomics in Precision and Personalized Medicine for Major Diseases

**Figure 1:**
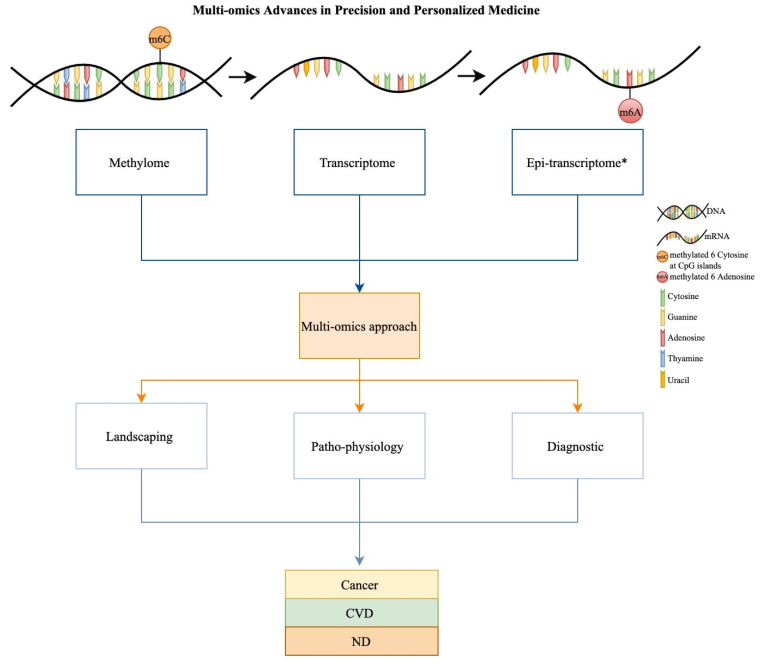
Multi-Omics advances in precision and personalized medicine.

The advent of genomic screens paved the way for the concept of precision medicine and personalized medicine (see Box 1) to be born. The greater part of the contribution has been from discoveries pertaining to the DNA sequence. While, thus, the centrality of genomics to precision medicine is well known and accepted, the contribution of transcriptomics is only recently being felt in the clinic. Here, we will discuss the impact of transcriptomics for major diseases via the lens of precision and personalized medicine initiatives (Figure 1). 

Schematic description showing the methylome, transcriptome and epitranscriptome relate to each other and how data sets from each may be used in concert for multi-omics investigations or used singly. Major advances using the above omics approaches for precision and personalized medicine efforts for cancer, cardiovascular disease (CVD) and neurodevelopmental disease (ND) have been yielded in disease landscaping, patho-physiological studies and diagnostics. *Epitranscriptomics data, especially the methylation of adenine at the 6 position of RNA, is particularly important in brain development and functioning, and hence in ND.

### 3.1. Cancer

The application of whole genome transcriptome profiling as a diagnostic tool has been proficient most in cancer [48,49]. Further, in the cancer diagnostic spectra, single cell transcriptomics and tissue-level transcriptomics, and now more recently spatial transcriptomics, have garnered great attention for their ability to potentially produce medically actionable results [50,51,52,53,54,55,56]. Transcriptomic profiling from single-cells conducted in concert with other ‘omics assays provide a multi-omic profile on cell lineage differentiation [51,57,58,59,60], and therefore, are particularly useful to trace tumor evolution, thereby producing gene-expression profiles actionable by specific drugs according to evolutionary stage [60]. These uses are highly specific in how they may contribute to diagnostics, and therefore, currently more prevalent in research settings [61], or settings where research occurs concomitantly with clinical diagnostics, such as within the Personalized Onco-genomics Project at the BC Cancer Agency [62]. However, given the rapid advance of technology plus concomitant plummeting costs, we anticipate it will likely enter mainstream diagnostics in the near future. 

Other than diagnostics, other notable contributions are advances in understanding tumor evolution and progress, especially if this may yield personalized/precision medicine treatment. Some examples are: application of RNAseq for earlier detection, and pinpointing aberrant metabolic pathways for ovarian cancer as reviewed by [63], in acute myeloid leukemia diagnosis and treatment [52,64], defining miRNA involvement in prostate cancer [50], and profiling of deregulated lncRNA expression as a marker for gastric cancer [65], and for cancer in general [56]. These are some examples of many studies and reviews focused on specific cancers, underscoring the immense strides transcriptomics is taking in cancer diagnosis and management. The interested reader may also refer to [60] and [53], [48] for general reviews of transcriptomics in cancer, and to [51] for an excellent review of onco-multi-omics.

### 3.2. Cardiovascular Disease (CVD)

Another disease where transcriptomics has made a significant mark is for CVD, considered one of the leading causes of death worldwide [66]. Similar to the situation in cancer research and treatment, transcriptomics-based advances in CVD are mainly two pronged: at the tissue and at the single-cell level, with the newly emerging spatial transcriptomics in hot pursuit. At the single-cell level, significant advances have been made in the past decade to trace, map and define evolution for every cardiac cell type [67]. Complete maps have been generated in murine cardiac tissue [68,69], and single cell RNAseq has proven useful to map the effect of myocardial infarction [70,71,72]. Work in our own species has just begun to emerge; In 2019, Cui et al. reported single cell RNAseq mapping for the development of the human heart during embryogenesis [73]. We anticipate these early publications to be among the vanguard of a rapidly maturing research field with great personalized and precision medicine potential.

A key contribution in CVD precision medicine is to understand tissue regeneration following insult. This will enable targeted treatments following major cardiac traumas. Therefore, several groups have focused on using single cell transcriptomics to better understand differentiation processes, inter-cellular signaling, as well as cellular pathway activation and modulation for cardiac tissue [74,75,76,77,78]. These studies are preliminary and are mostly confined to iPSC generated cellular models. Nevertheless, they are contributing vital landscaping at the single-cell level which is foundational for precision medicine efforts for CVD.

In contrast, tissue level transcriptomics has more direct diagnostic and therapeutic utility [79]. Efforts in this sphere have mainly included defining intrinsic and extrinsic risk factors for CVD, as well as determining personalized drug profiles for treatment; determining intrinsic risk at the transcriptome level involves defining an associated eQTL (expression quantitative trait locus/loci) signature for the heightened risk. The STARNET study published cardiometabolic risk loci determined by using RNAseq in 600 coronary artery disease patients [80]. Other notable efforts involve defining gene expression profiles produced by currently known genetic risk loci for CVD, as “transcriptome-wide association studies” [81,82,83], with the ultimate aim being able to better target drugs and accurately monitor drug response. However, these efforts are still confined to model systems [84,85] to our knowledge. A third relevant contribution is the ability to use transcriptome profiling to assess the impact of environmental changes on CVD [86]. Here, somewhat surprisingly, we note reports showing the adverse effect of noise pollution on vascular function, oxidative stress and resultant CVD risk [87,88], along with more expected studies showing health benefits of exercise (as witnessed by gene expression profiling) as an intervention to reduce CVD risk [89], and for impacts of diet and caloric intake on CVD [90,91]. Other notable achievements, especially from whole transcriptome RNAseq profiling studies, involve landscaping of ncRNA types in CVD [92,93,94,95], as early efforts to understand the impact of regulatory RNA on cardiovascular function and how cardiac tissue reacts to environmental stimuli.

### 3.3. Neurodevelopmental Disorders (ND)

After cancer and CVD, the third major health concern in Western countries, and one of the most common diseases globally is ND. ND affects between 1–3% of the global population [96], and constitutes a massive burden to health systems. Importantly, as ND affects children, the global disease burden includes immense psychological and other related difficulties faced by affected families and communities. Examining the impact of transcriptomics for ND as a whole is complex, as it encompasses a vast array of different disorders. They have in common that they manifest during childhood and involve developmental delays, usually including brain functioning, as well as multiple congenital anomalies. Intellectual disability (ID), and autism spectrum disorders (ASD) are the most well-known of the recognized NDs, and each of these itself constitutes an umbrella term under which dozens of different syndromes may be grouped [97]. 

The contribution of transcriptomics in the ND arena is currently not as robust as demonstrated for cancer or CVD. Nevertheless, the field is moving in the same direction as these two diseases, with concomitant efforts to map the transcriptomic landscape of the developing and functioning brain at single-cell [98,99,100,101,102] and macro [103,104,105,106,107,108,109,110] levels. Among the efforts to develop diagnostic RNA signatures in the developing brain, we draw attention to a review on evidence for causative lncRNAs [111], and tRNA metabolism [109] for general ND. We will briefly overview major research progress for the two major NDs, i.e., for ASD and ID next.

#### 3.3.1. ASD

ASD as a whole, though considered a complex genetic disease, has long suffered from a “missing heritability” problem [112], i.e., despite extensive genetic investigations, the initially expected full component of causative genes for the disorder have eluded detection. The application of transcriptomics to solve idiopathic ASD has yielded transformative insight into ASD pathogenicity and pathophysiology, that is, in addition to addressing the missing heritability quandary, revolutionizing how we understand the condition (as reviewed by [113]). The most significant contribution has been the revelation that autistic brains may possess a distinctive transcriptomic signature [113]; mRNA expression studies have shown changes in transcription levels for genes involved in neuronal function and immune response as well as spatial-specific profiles in examined brain tissue [114,115,116,117,118]. Further, aberrant gene transcription profiles have also been discovered in blood samples from children with ASD [119,120,121].

Similarly, specific studies looking at ncRNA have also provided evidence for possible brain profiles for ASD; miRNA profiles [122,123,124] and small ncRNA profile [125], lncRNA profiles [126] and even non-coding antisense transcripts [127] are reported. However, there is a startling lack of concordance amongst study findings [113], possibly attributable to the extreme variability inherent in transcriptomic experimental methods as well as within the ASD samples themselves. Nevertheless, the discordance is yet hampering efforts to obtain a profile that can be translated into true clinical significance.

#### 3.3.2. ID

The lack of consistently evidenced in ASD is exacerbated for transcriptomics in ID. It is more difficult to obtain a focused picture of where transcriptomics may be contributing for ID. Indeed, even where ID syndromes’ transcriptomes have been investigated, they are often in ID syndromes that include ASD as a co-morbidity. Examples are as follows: Rett syndrome [128,129], Pitt–Hopkins syndrome [130], and Zahir–Friedman syndrome [131,132]. In all these cases, transcriptomic profiling has been a means to investigate the pathophysiology of the known gene insult, but not to obtain a diagnostic transcriptome profile. 

In contrast to the work overviewed above, a very recent publication exemplifies investigations into using transcriptomics as a means of diagnosis for an idiopathic ND/ID disorder. A paper published a few months ago attempts to use the transcriptome to decipher causative signatures for the 3q29 deletion region that is a risk locus for ND [133]. While yet needing rigorous validation, this work highlights our anticipation that there will be more publications in the coming years that attempt to formulate a diagnostic role for transcriptomics in ND.

### 3.4. Other Diseases

We have briefly overviewed important contributions for cancer, CVD and ND produced by large scale and focused transcriptomic assays above. However, there are several other diseases that note revolutionary contributions. We are unable to adequately review them all due to space constraints and will instead collate highlights and focused reviews below.

Transcriptomics in concert with other multi-omics approaches is proving useful in unraveling the complex pathogenicity of; rheumatoid arthritis (as reviewed by [134]), of multiple system atrophy (a type of neurodegenerative disease, as reviewed by [135]), in diabetes and type-2-diabetes induced secondary health issues (as reviewed by [136,137]), in atherosclerotic CVD (as reviewed by [138]), for hypertension and its effects on multiple organ systems (as reviewed in [139]), and as diagnostic markers for preeclampsia (as reviewed by [140]). 

A call to use transcriptome profiling of neonatal saliva as an ideal means to assess infant development, as well as risk to a variety of disease, is also remarkable [141]. An idea that is being actively explored [142,143,144,145,146] and will certainly yield far-reaching precision and personalized medicine impacts if optimized to wide-spread clinical use. 

## 4. Epitranscriptomics for ND

When over-viewing transcriptomics contribution for ND, a specialized area that cannot be ignored is the emerging “epitranscriptomics” which is rapidly gaining traction as significant, due to findings specific to the brain’s development and function (Figure 1). We will briefly address major epitranscriptomics advances in ND next. We begin with a concise definition and explanation.

### 4.1. The Epigenome and the Brain 

To understand the epitranscriptome, we must first introduce and summarize the methylome and methylation. Methylomics is the study of patterns of methylation, usually genome wide. We have extensively discussed methylation as part of epigenetics/epigenomics, and its contribution to ND in other reviews [147,148]. There have been many recent publications focused on the impact of methylation in ND, however, similar to the situation discussed above with respect to transcriptomics, they predominantly report mapping efforts for causative methylation profiles due to known genetic insults [149,150], rather than as true stand-alone diagnostic markers. As we have previously covered this topic in depth [148], and as more recent reviews focus on it [149,150,151,152], we will not delve into it further here. 

In terms of the assays used for methylomics screening, they are the same as for genomics, except that they differ in how the sample material is prepped prior to loading onto the assay of choice; either CMA or NGS. Briefly, the initial DNA sample is subjected to isolation methods that will specifically pull-down methylated sequences. These are then de-methylated and subjected to sequencing methods as usual [98]. Interestingly, the same procedure can be applied to cDNA samples originating from mRNA, and thereby it is possible to obtain profiles of methylation on mRNA. This is precisely the premise of epitranscriptomics assays. Apart from methylation of mRNA, several other epigenomic signatures are known for many types of RNA [153,154]. Here we only cover a specific epitranscriptomic signature that appears to have disproportionately great impact on brain development and functioning.

### 4.2. Epitranscriptomes in the Brain: m6A RNA Modifications in ND

Epitranscriptomics or “RNA epigenetics” refers to the study of post-transcriptional chemical modifications of RNA. Over 150 RNA modifications are currently known [153,155]. Though several RNA species may be “epi-modified”, among them; mRNAs, tRNAs and miRNAs [154], to our current knowledge, mRNA methylation accounts for the major faction [156]. Methylation of adenine at the 6 position (m6A) of RNA is frequently found in brain tissue [155,157], and is garnering increasing attention as important in brain development, function and plasticity [for reviews see [156,157,158,159,160]. As elegantly reviewed by Shafik and colleagues, dynamic m6A RNA methylation is being shown to play a pivotal role in how the brain responds to environmental impacts and in the development of disease [157]. Indeed, an emerging field of “environmental epitranscriptomics” is showing how epigenetic modification of RNA in response to changes in the surrounding environment can impact a variety of biological processes [153]. While this field is yet in its infancy and not robust enough to significantly influence precision/personalized medicine, we anticipate a future impactful role from epitranscriptomics especially for ND [161], as the brain seems to be particularly susceptible to epitranscriptome signaturing.

## 5. Conclusions

In this paper, we have overviewed major achievements for transcriptomics and epitranscriptomics in precision and personalized medicine. Important to remember is that transcriptomics (and epitranscriptomics) assays often are generated and indeed work best when they are analyzed in the context of “other-omics” or more correctly, “multi-omics” [10,51,60,137], as the ability to layer several maps of data present a complete picture of genomic activity and resulting biological impact (Figure 1). Often transcriptomics assays are conducted in concert with methylomics assays. The results of each serve to validate the other. For example, a methylation profile indicating a genome-wide gene on-off signature should ideally be validated by a genome-wide gene expression profile that matches it, and vice versa. This is why several of the transcriptomic studies referenced in this paper are often presented in the context of “multi-omic” studies. However, an obvious detriment to such robust screening is the rising cost of layering genome-wide “omics” screens. Hence, it is important to discuss the clinical utility of transcriptomics assays as stand-alone tests. 

As we have reviewed, in the short time since transcriptomics screening has arrived on the main stage of “omics in precision and personalized medicine, it is already making significant inroads in some diseases. Notable are the efforts in cancer, CVD and to some extent, in ND (especially for ASD, when considered in concert with epitranscriptomics). The extensive citations we have been able to collate in this paper are by no means exhaustive, showcasing the breadth and depth of contribution. When taken as a whole, we note that advances are being made on three major fronts; (a) landscaping the normal and diseased tissue states, (b) developing accurate diagnostic profiles that are both personalized and precise, and (c) investigating environmental interventions’ impact including for introduced therapies (Figure 1). Each of these areas is inherently rich in potential for discovery and innovation that will have real-life clinical impact. 

We have added a section dealing with the very recent but no less impactful field of epitranscriptomics, and its special place when speaking of the development and function of the human brain, and therefore by corollary, for ND. As early results show, there is a lot more to be discovered as to how specific post-transcriptional modifications that are so prevalent in the human brain, lends to both normal functioning as well as disease causation. One aspect that seems certain to-date however, is that the brain epitranscriptome is mechanistically significant as part of the processes that yield to the essential and remarkable plasticity of the brain. 

Our review of the existing literature may appear to focus on diagnostics as opposed to therapeutics. While we have noted therapeutic inroads for cancer and CVD, such efforts for ND are yet not realized. Even the advances we note for cancer such as the Personalized Onco Genomics project [62] are instances where precision tumor drug targeting has been possible when transcriptomic profiling is carried out in concert with major diagnostic landscaping and probing efforts. In the CVD domain, therapeutic efforts to-date predominantly align with identifying environmental and behavioural risk [79,80,81,82,83,84,85,86,87,88,89,90,91], and we surmise, therefore, that therapeutic efforts follow upon mitigating risk or risk reduction via life-style interventions. The third of the major diseases discussed here, ND (and autism in particular), is lagging in the domain of precision therapeutics informed by transcriptome or epitranscriptome profiling. ND is a highly complex, multi-faceted, heterogenous disease group for which precision therapeutics based upon omic profiling has still not yet reached advances robust enough to review. 

Nevertheless, as costs reduce and databases of transcriptome profiles grow in size, depth, and breadth, as well as in data reliability and rigor, it will become increasingly easier to generate and interpret transcriptomic datasets. This positive research cycle we expect to definitively yield notable, and we hope far reaching, precision and personalized medicine advances, that will span diagnostics, prophylaxis, and therapeutics.

## Data Availability

Not applicable.
